# Corn360: a method for quantification of corn kernels

**DOI:** 10.1186/s13007-023-00995-2

**Published:** 2023-03-09

**Authors:** Samantha Gillette, Lu Yin, Penny M. A. Kianian, Wojciech P. Pawlowski, Changbin Chen

**Affiliations:** 1 School of Life Sciences, Arizona State University, Tempe, AZ 85287 USA; 2 PepsiCo Inc., 210 Borlaug Hall, 1991 Upper Buford Circle, St. Paul, MN 55108 USA; 3 School of Integrative Plant Science, Cornell University, Ithaca, NY 14853 USA

**Keywords:** Image analysis, High-throughput phenotyping, Kernel color, Kernel texture, Low-cost, Corn

## Abstract

**Background:**

The rapidly advancing corn breeding field calls for high-throughput methods to phenotype corn kernel traits to estimate yield and to study their genetic inheritance. Most of the existing methods are reliant on sophisticated setup, expertise in statistical models and programming skills for image capturing and analysis.

**Results:**

We demonstrated a portable, easily accessible, affordable, panoramic imaging capturing system called Corn360, followed by image analysis using freely available software, to characterize total kernel count and different patterned kernel counts of a corn ear. The software we used did not require programming skills and utilized Artificial Intelligence to train a model and to segment the images of mixed patterned corn ears. For homogeneously patterned corn ears, our results showed accuracies of 93.7% of total kernel count compared to manual counting. Our method allowed to save an average of 3 min 40 s per image. For mixed patterned corn ears, our results showed accuracies of 84.8% or 61.8% of segmented kernel counts. Our method has the potential to greatly decrease counting time per image as the number of images increases. We also demonstrated a case of using Corn360 to count different categories of kernels on a mixed patterned corn ear resulting from a cross of sweet corn and sticky corn and showed that starch:sweet:sticky segregated in a 9:4:3 ratio in its F2 population.

**Conclusions:**

The panoramic Corn360 approach enables for a portable low-cost high-throughput kernel quantification. This includes total kernel quantification and quantification of different patterned kernels. This can allow for quick estimate of yield component and for categorization of different patterned kernels to study the inheritance of genes controlling color and texture. We demonstrated that using the samples resulting from a sweet × sticky cross, the starchiness, sweetness and stickiness in this case were controlled by two genes with epistatic effects. Our achieved results indicate Corn360 can be used to effectively quantify corn kernels in a portable and cost-efficient way that is easily accessible with or without programming skills.

**Supplementary Information:**

The online version contains supplementary material available at 10.1186/s13007-023-00995-2.

## Background

The rapidly advancing plant breeding field calls for high-throughput methods and technologies for phenotyping such as Artificial Intelligence (AI) assisted image analysis. Corn is the number one ranking crop for production with 93 million acres grown in the United States in 2021 [1], where tremendous breeding efforts have been made to improve corn yield, drought tolerance, and nutritional values [2]. Kernel traits, as components of yield, have been traditionally evaluated manually, which are labor-intensive, time-consuming, low efficient, and can be error prone [3]. Yet, the idea of technology-assisted image analysis is not new. There are several existing methods that phenotype corn kernel traits in a high-throughput manner [3–6]. Particularly, panoramic photography of corn ears is useful to obtain information from the whole ear and avoids biases arising from a partial view. Available methods to capture panoramic images of corn ears often requires a sophisticated, stationary setup such as a rotating frame to collect the image data and multiple steps of transformation and/or algorithms to process and analyze the images [7, 8]. We aimed to develop an imaging system that is affordable and does not require much programming or statistical expertise. The availability and accessibility of such technology globally across corn grown environments at developed and developing countries is beneficial.

Such panoramic, high-throughput, cost-effective, easily accessible methods not only assist in phenotyping for plant improvement, but also assist in furthering our understanding of genetic inheritance of certain traits. This is particularly true to study genes that contribute to the white, waxy, sweet, popcorn, high-amylose, high oil, and quality protein attributes in different types of corn [9]. For example, the yellow, red, and purple kernels were found to be controlled by four genes and produced by pigments synthesized in the carotenoid and anthocyanin pathways, while the white kernels are a result of a lack of pigments produced from either pathways [10]. The inheritance of sweet and sticky corns seems to be more complex (see Additional file 1 for examples of dried sweet corn which are yellow, shriveled and translucent, starch corn which are yellow and opaque with a translucent appearance and sticky/waxy corn which are yellow and opaque). There can be up to three sweet genes, where one or two are mostly responsible for the sweetness in traditional sweet corn [11]. A recessive mutation of one of the sweet genes is responsible for sticky kernels, while other sticky genes can exist in the background of the sweet genes [11, 12]. To be able to quantify the segregation ratio in a sweet corn × sticky corn cross would offer more insights into the genetic relationships of these genes (Additional file 2).

We used the panoramic images taken by a smart-phone and a turntable and quantified corn kernels through the freely available Food Color Inspector and ImageJ processing program on a PC computer (or any system where Java 8 runtime is available) [13]. The smart-phone or any other smart devices such as iPad or Tablet which would work equally sufficient and are easily accessible to an average researcher with or without programming skills. The display turntable used for image collection is powered by batteries, small-sized, and costs ~ $20, which makes it portable to be used in the field. The Corn360 system is an inexpensive, portable system ideal for AI counting of corn kernels which can be used to characterize the kernel counts of total kernels and different colored/patterned kernels. The Corn360 system can be easily incorporated into existing breeding programs with other more complex image analysis toolsets and be used as tools for teaching in genetic classrooms.

## Results

**Fig. 1:**
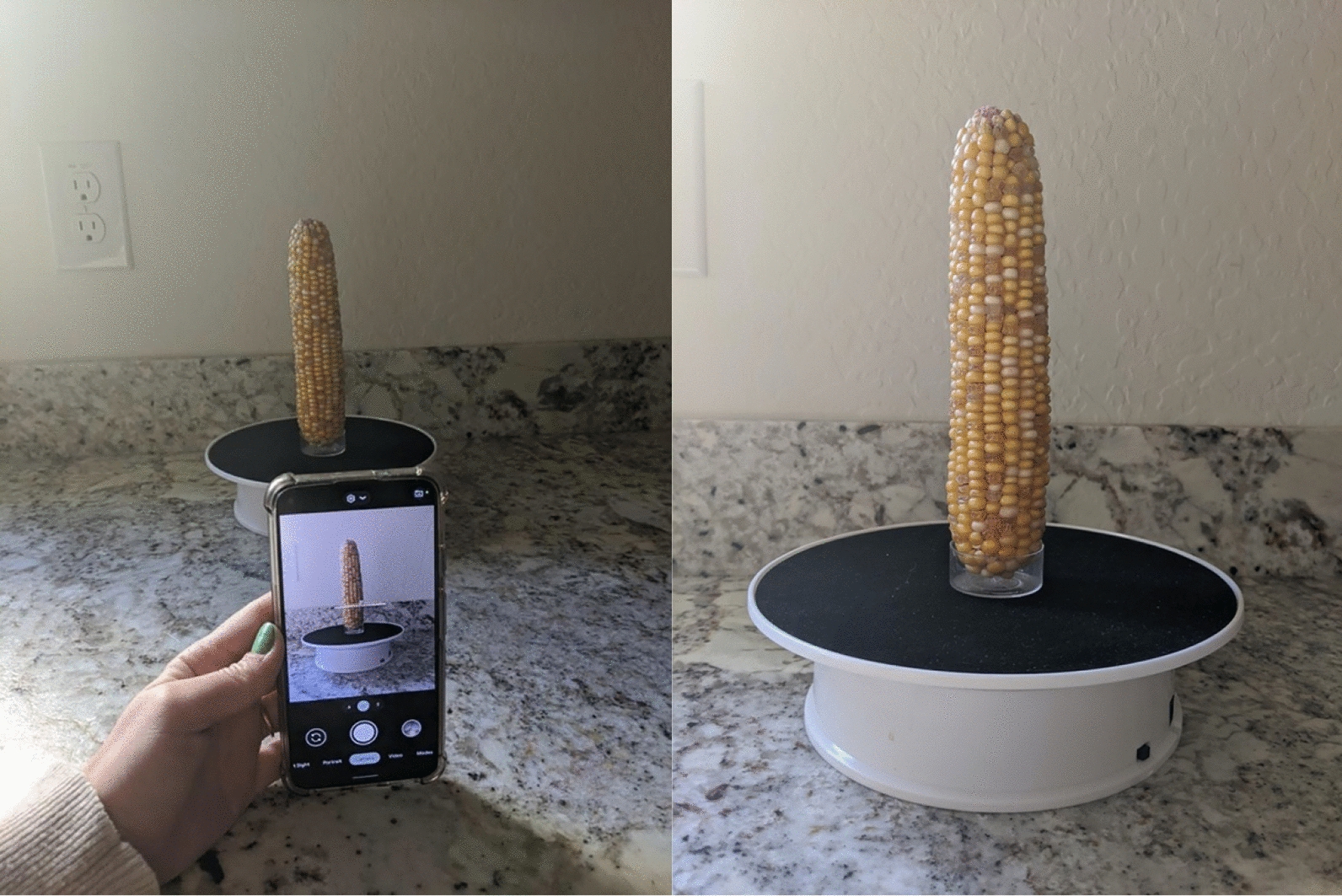
The Corn360 system. Panoramic image capturing as the corn ear revolves (L) and A corn ear is placed on the turntable (R). *L* left, *R* right

**Fig. 2:**
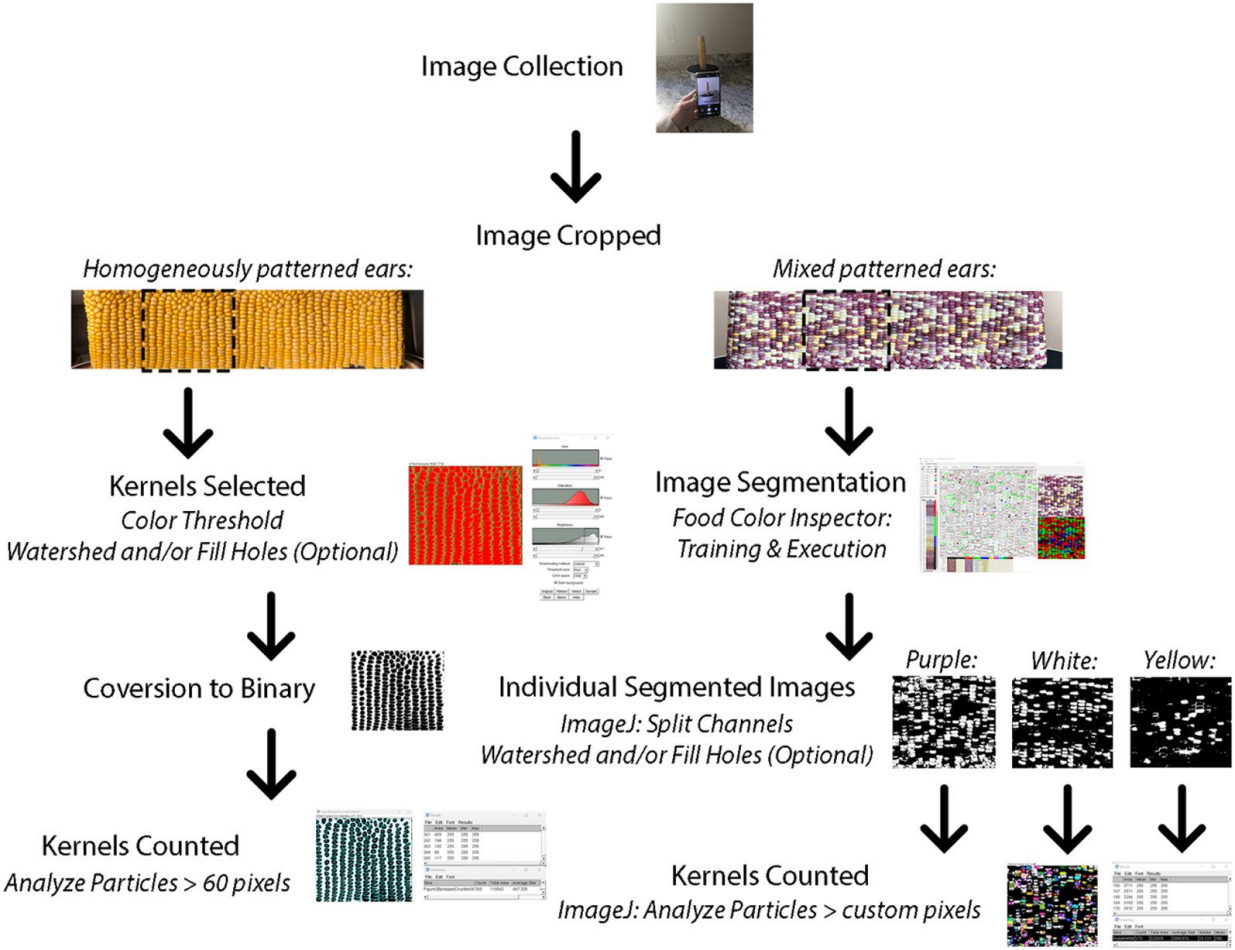
Image processing steps for homogeneously patterned and mixed patterned corn ears

The Corn360 image collection system consists of a smart phone and a battery-powered turntable (Fig. 1; see “Methods”). The panoramic image is taken while the corn ear rotates at least one rotation on the turntable. The image is then prepared for image analysis to include exact one full rotation of the ear. Here we demonstrate the use of this Corn360 system combined with subsequent image analysis to count kernels (total kernel and/or different categories of kernels) on three different cases of corn samples: homogeneously patterned corn ears, mixed colored corn ears, and mixed textured corn ears (Fig. 2).

**Fig. 3:**
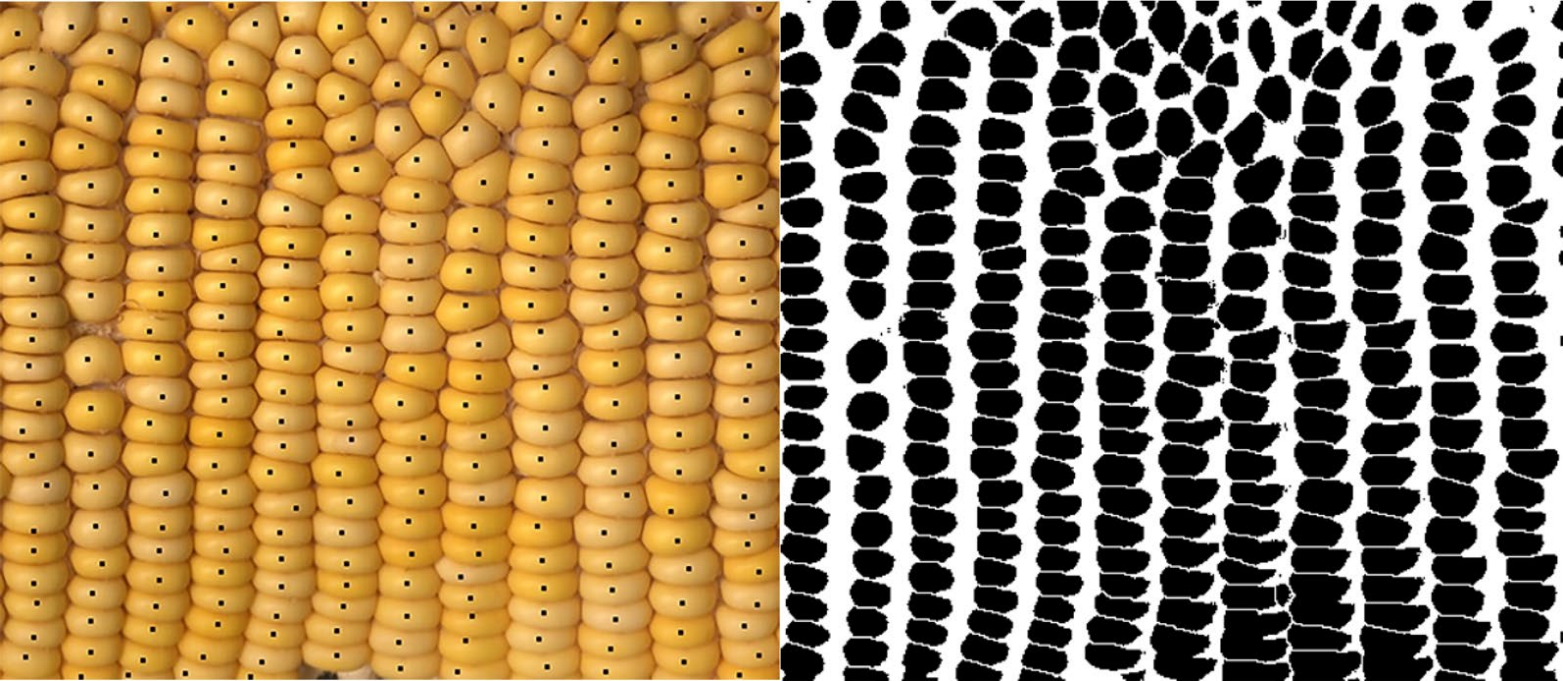
An example of total kernel count of a homogeneous patterned corn ear: Manual count of total number of kernels (L) and ImageJ count of total kernels (R)

For corn ears with homogeneously patterned kernels, the total kernel count is obtained via manual count (Fig. 3) versus the “AI” ImageJ Analyze Particle count on a black and white (binary) image (Figs. 2, 3). The ImageJ method showed an average accuracy of 93.7% compared with manual count (Table 1). The AI method takes an average of 33.75 s per each sample while manual count takes 4 min 13 s per sample, saving about 3 min 40 s per image (Table 1).

**Table 1:** Total kernel count of different sample corn ears using the ImageJ binary analysis count and manual count

Corn ear samples	Replicate	Kernel count		%Accuracy	Counting time	
		Manual	ImageJ AI		Manual	ImageJ AI^a^
Red	1	423	421	99.5	4 min 2 s	17 s
Red	2	611	602	98.5	5 min 27 s	1 min 2 s
Yellow	1	269	265	98.5	4 min 32 s	54 s
Yellow	2	264	321	78.4	2 min 51 s	2 s

^a^The time it takes to run and modify the macro, not including the time to troubleshoot when the macro goes wrong, which can take up to 10 min

**Fig. 4:**
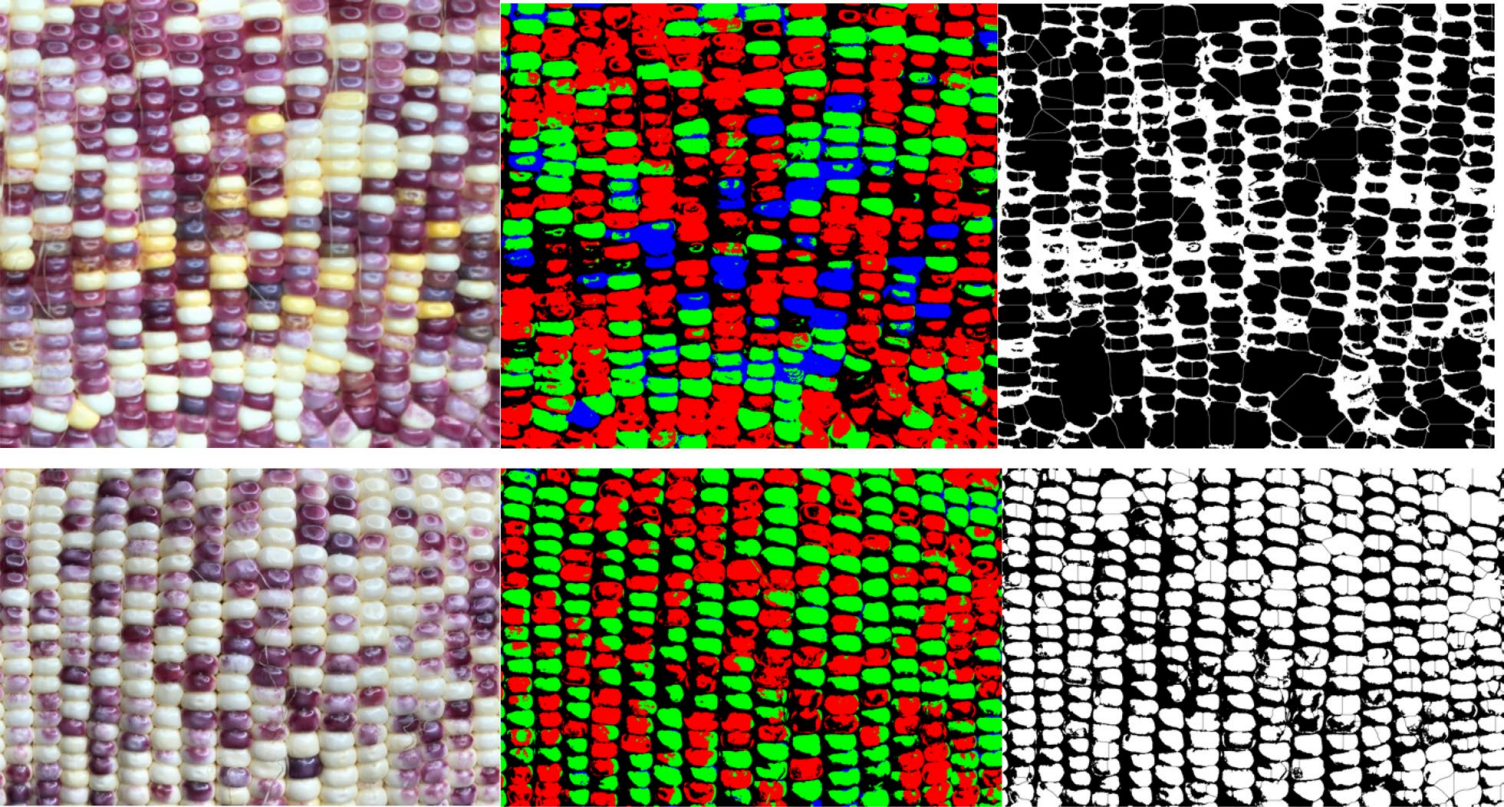
Mixed colored corn ear sample 1 (upper panel) was used to train the Food Color Inspector: Original image (L), segmented image (M), and adjusted binary image (R) showing total kernels (black pixels), as well as mixed colored corn ear sample 2 (Lower panel) where the training was executed: the original image (L), segmented image (M), and adjusted binary image (R) showing total kernels (white pixels). *L* left, *M* middle, *R* right

**Fig. 5:**
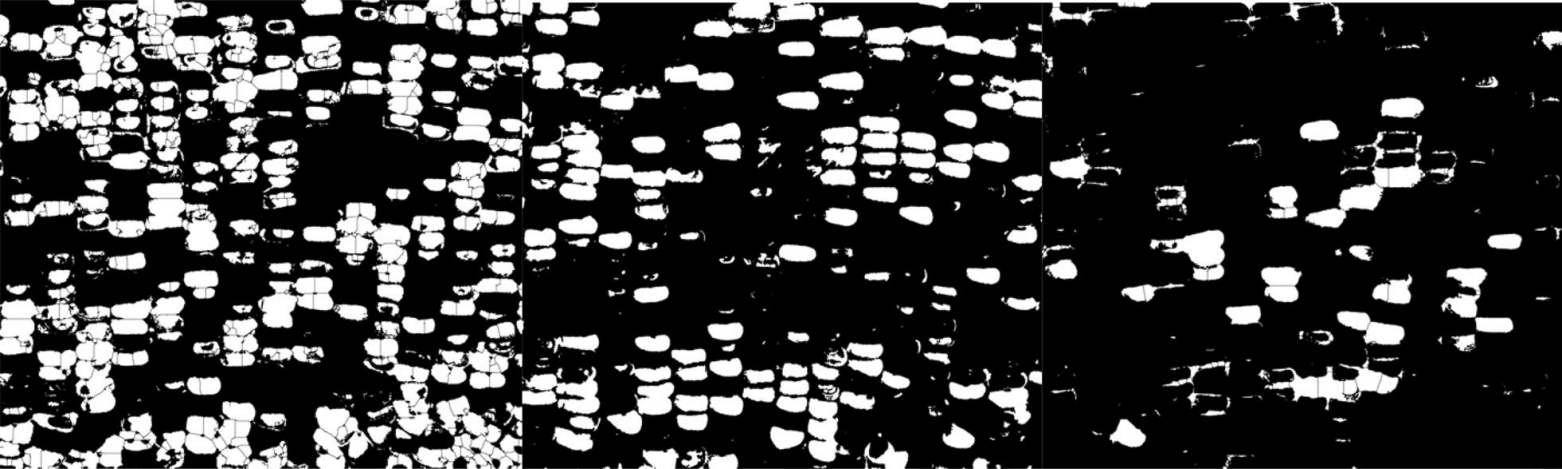
An example shows the Food Color Inspector segmented split channel adjusted binary images for the classes purple (L), white (M), and yellow (R) kernels of the mixed colored corn sample 1 shown in Fig. 4

For corn ears of mixed colors, the image is first segmented by Food Color Inspector (see “Methods”) by classifying examples of selected categories and then this trained module is saved and applied to different images (Figs. 2, 4). These data are subsequently split into three channels of binary images coupled with the Analyze Particle function to quantify segmented kernel counts of purple, white, and yellow classes, whereas total kernel count is obtained via the binary image before channel splitting (Figs. 2, 5; see “Methods”). The accuracy for total kernel count was 85.9% and average accuracy for segmented kernel counts was 84.8%, where that for purple, white, and yellow kernels was 85.4%, 87%, and 79.3% respectively (Table 2). The AI method takes an average of 2 min 37 s per category per sample, slightly longer than manual counting which takes 2 min 3 s (Table 2).

**Table 2:** Total and segmented kernel count of mixed colored corn ear sample 1 as shown in Fig. 4 used to train the classifier of the Food Color Inspector and total and segmented kernel count of mixed colored corn sample 2 where the classifier was executed

Kernel	Sample	Kernel count		%Accuracy	Counting time	
		Manual	AI		Manual	AI^a^
Total	1	381	285	74.8	3 min 21 s	5 min 51 s
Purple	1	238	175	73.5	3 min	2 min
White	1	103	83	80.6	1 min 41 s	3 min
Yellow	1	29	23	79.3	55 s	1 min 50 s
Total	2	301	310	97	2 min 24 s	2 min 20 s
Purple	2	142	146	97.2	1 min 27 s	2 min 33 s
White	2	151	141	93.4	1 min 33 s	48 s

^a^The time it takes to train and run the Food Color Inspector, image adjustment, and Analyze Particle, not including the time to troubleshoot the best image adjustment steps, which can take up to 10 min

**Fig. 6:**
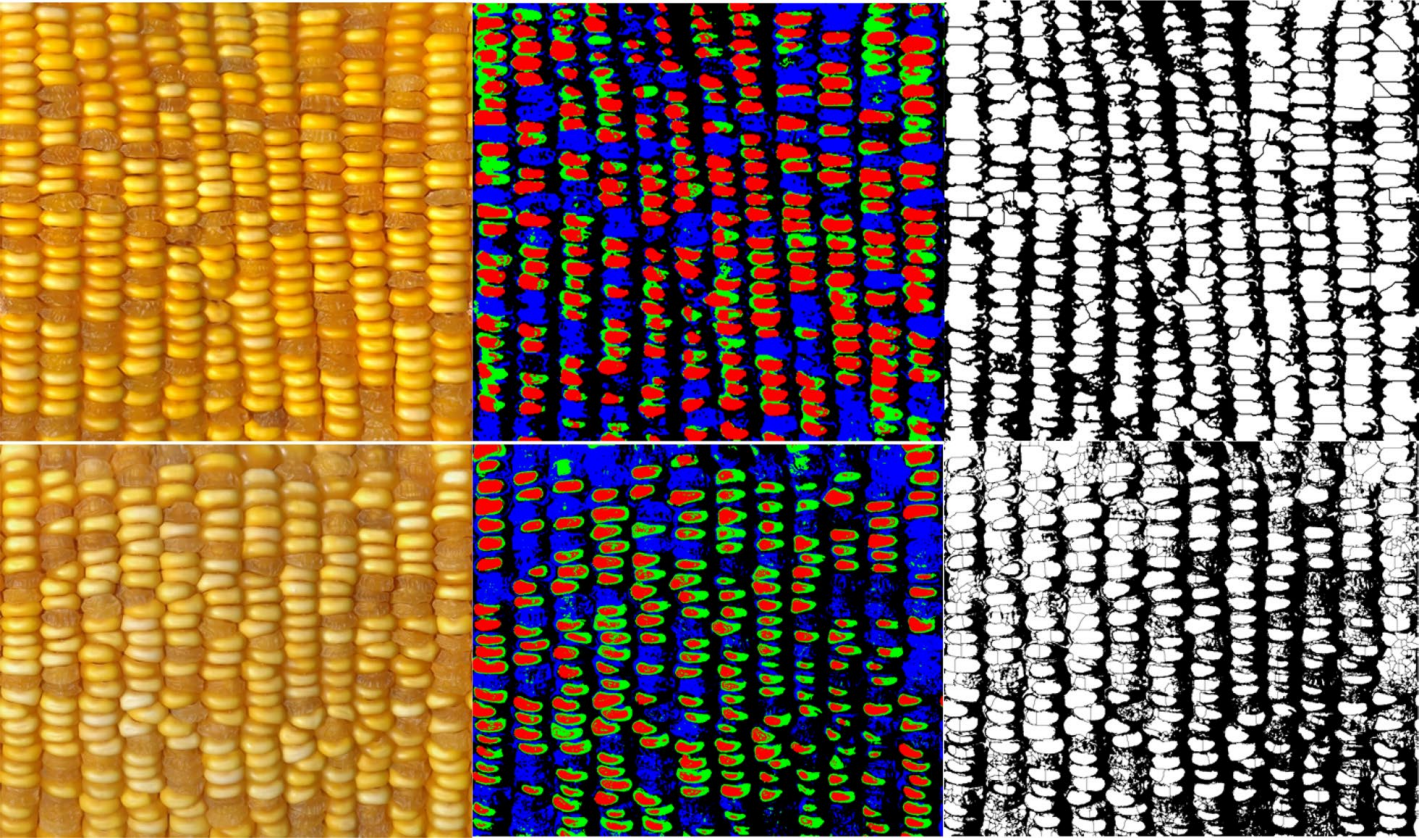
Mixed textured yellow corn sample 3 of different texture (also shown in Additional file 2) was used to train the Food Color Inspector (upper panel): Original image (L), segmented image (M), and adjusted binary image (R) showing total kernels; and mixed textured yellow corn ear sample 4 (lower panel) where the training was executed: the original image (L), segmented image (M), and adjusted binary image (R) showing total kernels

**Fig. 7:**
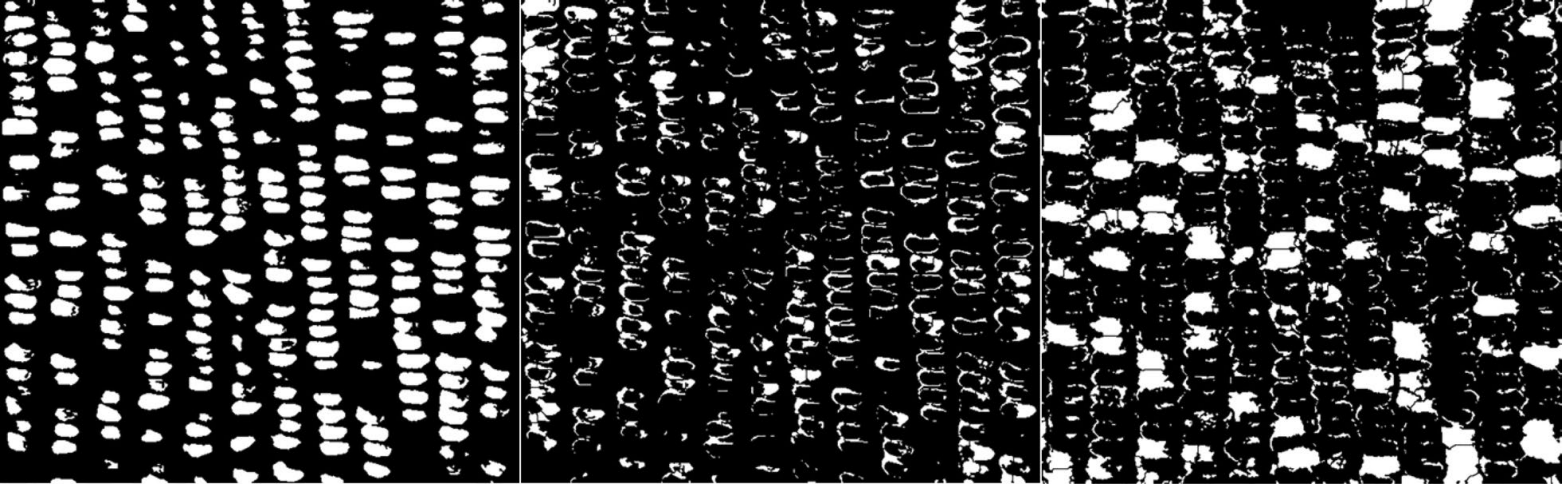
An example shows the Food Color Inspector segmented split channel adjusted binary images for the classes sticky (L), starch (M), and sweet (R) kernels of the mixed textured corn sample 3 shown in Fig. 6

This segmentation method can also be used to segment a corn ear with kernels of different textures as in sweet × sticky hybrid samples (Figs. 6, 7, Additional file 2). The average accuracy for counting total kernels was 93.9%, for segmented kernel count was 61.8%, where that for sweet kernels was 79.6%, for sticky and starch kernels was 28.5% and 77.4% respectively (Table 3). The average manual counting time is 2 min 30 s, while AI counting time is 1 min 55 s, saving 35 s per image (Table 3).

**Table 3:** Total and segmented kernel count of mixed textured yellow corn ear sample 3 as shown in Fig. 6 used to train the classifier of the Food Color Inspector and total and segmented kernel count of mixed textured yellow corn sample 4 where the classifier was executed

Kernel	Sample	Kernel count		%Accuracy	Counting time	
		Manual	AI		Manual	AI^a^
Total	3	341	307	90	2 min 52 s	4 min
Solid opaque (sticky)	3	65	98	49.2	3 min 50 s	2 min
Shiny opaque (starch)	3	175	101	57.7	3 min 54 s	1 min 25 s
Shriveled translucent (sweet)	3	85	64	75.3	51 s	53 s
Total	4	318	311	97.8	2 min 41 s	2 min 18 s
Solid opaque (sticky)	4	39	75	7.7	2 min	2 min 17 s
Shiny opaque (starch)	4	170	175	97.1	2 min 43 s	1 min 2 s
Shriveled translucent (sweet)	4	87	73	83.9	1 min 6 s	1 min 28 s

^a^The time it takes to train and run the Food Color Inspector, image adjustment, and Analyze Particle, not including the time to troubleshoot the best image adjustment steps, which can take up to 10 min

For total kernel count on mixed patterned corn samples, the ImageJ method (binary image with particles counted by Analyze Particle) showed an average accuracy 24.9% (data not shown), while the AI method (binary image from Food Color Inspector-segmented image with particles counted by Analyze Particle) showed an accuracy of 85.9% (Table 2). Thus, for mixed patterned samples, it is recommended to use the Food Color Inspector to first segment and then count the total kernels of the binary image, instead of counting that of the binary image directly.

As an example of using the system to study genetic inheritance, we used the sweet × sticky hybrid sample 3 (Figs. 6, 7, Table 3) and conducted a Chi-square test to test for the segregation ratio, where $\upchi ^{2} = \sum {\frac{{\left( {Observed - Expected} \right)^{2} }}{Expected}}$. The hypothesis is that starch:sweet:sticky kernel count fits a 9:4:3 ratio. In other words, if the hypothesis is true, the starchiness, sweetness, and stickiness of the kernels in this sample are controlled by two genes with epistatic effects. $\upchi ^{2} = \frac{{\left( {175 - 191.81} \right)^{2} }}{191.81} + \frac{{\left( {85 - 85.25} \right)^{2} }}{85.25} + \frac{{\left( {65 - 63.94} \right)^{2} }}{63.94} = {1}.{49}$ with degrees of freedom of 2. This 1.49 value is smaller than the Chi-square value at alpha 0.05 which is 5.99. Thus, we failed to reject the hypothesis that the starch–sweet–sticky sample fits a 9:4:3 ratio.

## Discussion

The Corn360 system with a simple setup consisting of a smart-phone and a turntable offers great accessibility and affordability for average researchers (without programming or statistical expertise) and professionals to efficiently capture panoramic images of corn ears with relatively high accuracy. It was demonstrated to obtain a total kernel count of homogeneously patterned corn samples with high accuracy (93.7%) and to obtain kernel counts of different categories of mixed patterned corn samples with relatively high accuracies (84.8% or 61.8%). As of now, the method outlined saves about 3 min 40 s per image of counting total kernels of a homogenously patterned corn ear compared with manual counting and has the potential to save a vast amount of time to count segmented kernels of a mixed patterned corn sample, as the number of images increases.

We have demonstrated an example of the use of segmented kernel counts identified by Corn360 method to study trait segregation and demonstrated that a corn sample with mixed starch:sweet:sticky kernels was segregating in a 9:4:3 ratio. This suggests that the starchiness/sweetness/stickiness of the kernels are controlled by two genes with epistatic effects. A dominant copy at both genes (A_B_) are required to have starch kernels, a dominant copy at the first gene but not the second gene (A_bb) results in sticky kernels, no dominant copy at the first gene (aaB_ and aabb) results in sweet kernels. The gene A we identified is very likely *Sugary1*. Recessive *sugary1* mutations have been shown to be responsible for sweetness in traditional sweet corn, where the *Sugary1* gene is responsible for amylopectin crystallinity and biosynthesis that is important for sticky corn [12]. A second gene B seems to be important for starch synthesis, where gene A plays an epistatic role, such that gene B needs gene A to produce starch and without gene A, a dominant copy of gene B still results in sweet kernels. This information helps us better understand the inheritance of these genes in our sweet × sticky germplasm, which can benefit us in making informed breeding decisions in the future and can also be used as a case study to teach Mendelian genetics and epistasis.

Corn360 utilizes image processing software that do not require programming or statistical expertise. Because of this, Corn360 remains semi-automatic and its accuracy and counting time are limited. This is particularly true for adjacent kernels that do not separate well and kernels that have similar colors or textures. For example, for counting the sweet corn kernels, the accuracy was not very high mostly because of the challenge to differentiate the adjacent sweet corn kernels which had the same color with no clear gaps between them that can be recognized as the background class. Similarly, the accuracies for sticky and starch kernel counts were relatively low due to the subtle differences between them. Because the program recognized starch kernel shiny outer layer as one class but could not differentiate the interior from sticky kernels (Fig. 6), a lot of tweaking needs to be done on particle size selection to differentiate and count these kernels. We tried incorporating other software tools to enhance kernel edges such as Color Deconvolution [14] into our current pipeline, but not much improvement obtained. Sometimes the seemly accurate ImageJ/AI counts were a result of Watershed-separating the kernels at the wrong place and the counts happened to be close to the manual counts. It seems the best solution to such issues and/or to further improve accuracy requires the integration of programming-based image analysis pipelines [3, 15]. The accuracy also increases when there is a less dense sample with kernels. In the particular case of hybrid corn sample 4, the clearly defined outer shiny layer allowed the Fill Holes function of ImageJ to fill in the starch kernels and gave a highly accurate starch kernel count. After all, Corn360 is a quick and direct method to assess kernel counts in a portable way for average breeders who may or may not have the programming and/or statistical expertise.

The method presented is a semi-automatic method where manual image adjustment is still needed on different corn samples. We outlined several parameters that are recommended to be kept constant while a few parameters that need to be adjusted for each different sample. Firstly, it is recommended to train the classifier more precisely by selecting more examples of the gaps between these kernels as background, particularly when ImageJ has a hard time to differentiate adjacent same patterned kernels. Secondly, it is recommended to take all images at the same distance to the corn ears, under the same illumination, to avoid much of the manual tweaking of the image analysis process. With these parameters kept constant, once the image in cropped, the places that need to be adjusted for each homogeneously patterned corn ear are the Kernel Selection step for Watershed and Fill the Holes functions, and Color Threshold values, and the Kernel Counting step for particle size to be counted (Fig. 3; Additional file 4). In addition to adjustment of the image processing and counting steps (Additional file 5), different patterned ears need to be trained anew using the Food Color Inspector. It is recommended to train the classifier on the corn ear with the most patterns and apply it on corn ears with any subset of the patterns. For example, the classifier trained using a corn sample of purple, yellow, and white kernels was successfully applied to and segmented a corn sample with purple and white kernels. Sometimes, the macro needs to be executed in separate steps in order to run properly.

More specifically, we discuss a few limitations and potential solutions. Future research should look into this to make it more accurate perhaps through automating this process using programming scripts from previous studies As of now, this Corn360 method presents an inexpensive method for breeders and researchers without non-programming skills. Potential solutions include enhancement of the kernel edges using the color deconvolution algorithm or the connected domain analysis [4, 8].

The total number of kernels is an important component of yield and Corn360 presents a low-cost way for corn breeders to screen through hundreds and thousands of varieties efficiently. Besides total kernel and segmented kernel counts, other traits such as corn ear length, radius, row number, kernel area, and kernel size, as well as those similar to grape cluster traits such as aspect ratio (corn ear radius/length) and compactness can also be efficiently extracted using the Corn360 panoramic imaging capturing system [8, 15]. With equipment modifications such as a rotating trunk mounted tripod or an aerial drone, the use of Corn360 can be extended to other cylindrical objects such as fruits and tree trunks for non-destructive assessment for diseased spots, growth rate, or bark pattern (Additional file 3).

## Conclusions

Corn360 has a simple setup and is portable, easily accessible, and affordable. The simple setup which requires a smart phone with a panoramic camera function, a turntable, and the freely available image processing software installed on a computer. We have demonstrated its accuracy and efficiency in counting total kernels of a homogeneously patterned corn ear and its potential to be used to count segmented kernels of a corn ear with mixed patterned kernels. It is a semi-automatic method and can batch-process numerous images with custom ImageJ macro scripts and the trained Food Color Inspector classifier. With different samples, depending on its kernel density, the classifier and the macro scripts need to be manually adjusted accordingly. Corn 360 setup is also portable which enables for panoramic images to be taken and prepared in real-time at the field. The results demonstrated that Corn360 AI counting can maintain a relatively high accuracy compared to manual counting with the potential to save vast amounts of time for corn breeders and researchers who may or may not have the programming and statistical expertise. Corn360 can be readily incorporated into breeding programs and/or genetic classrooms.

## Methods

### Image collection

#### Preparation of the Corn 360 image collecting system

Corn360’s collection system consists of a battery-operated turntable (also has a power cord for stationary uses) with a 20-cm diameter paired with a device to support a corn ear on the platform. The turntable was purchased from Amazon for ~ $20. In Fig. 1, a clear plastic candle holder is used to avoid blocking any kernels of the corn ear. A screw can be used to achieve the same goal. Once the corn ear is securely placed, a smart phone is used to take a panoramic image. To take the image, the smart device/phone is held still (or with a mini tripod) while the turn table turns at 10 s/revolution for at least one revolution (Fig. 1). This small area and battery powered equipment allowed for it to be easily portable for off-site locations.

#### Preparation of photos for ImageJ segmentation

**Fig. 8:**
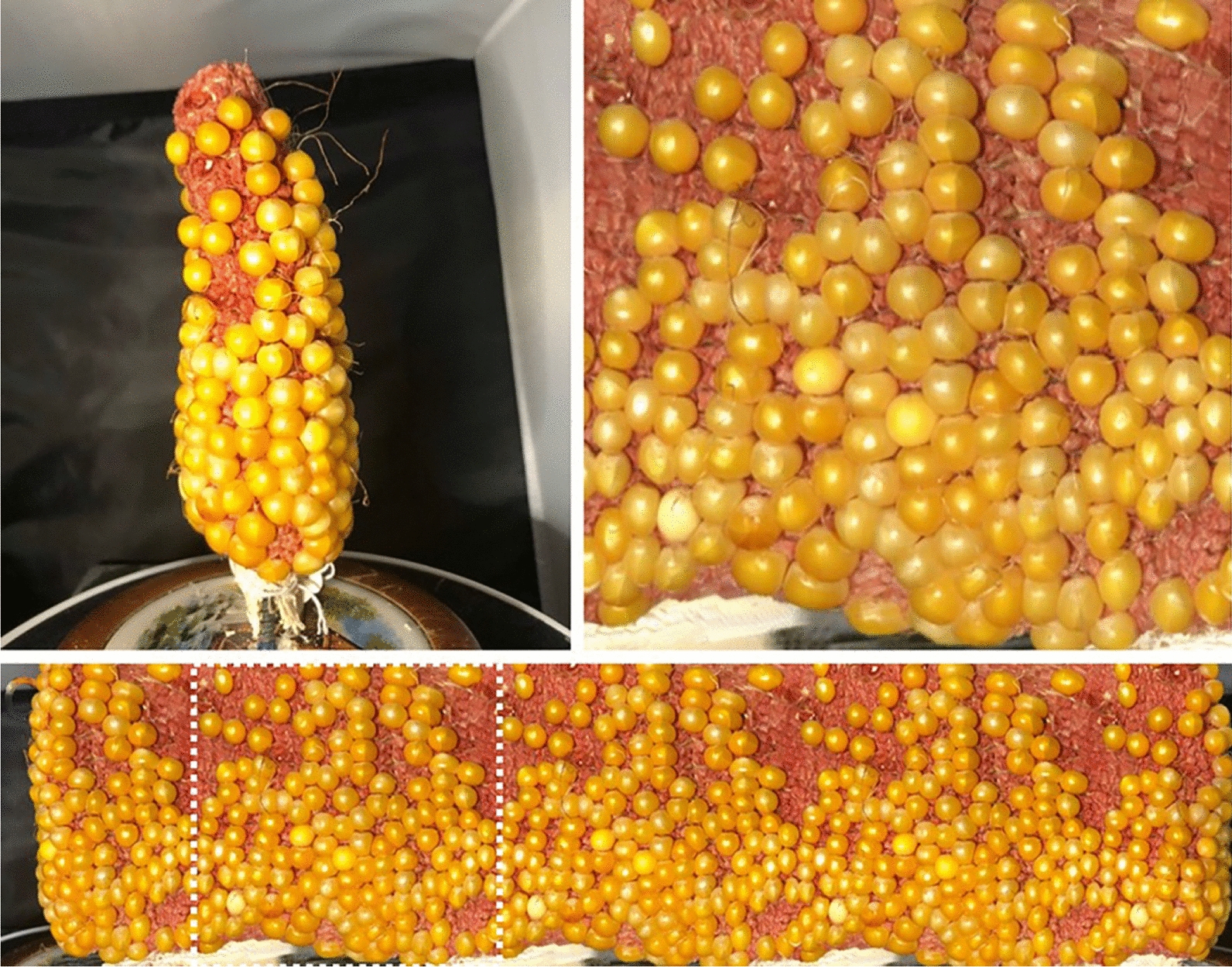
An example of fully prepared panoramic image with one full revolution using a landmark. The corn ear (upper L), one full revolution (upper R), and the raw panoramic image with one revolution marked (lower panel)

**Fig. 9:**
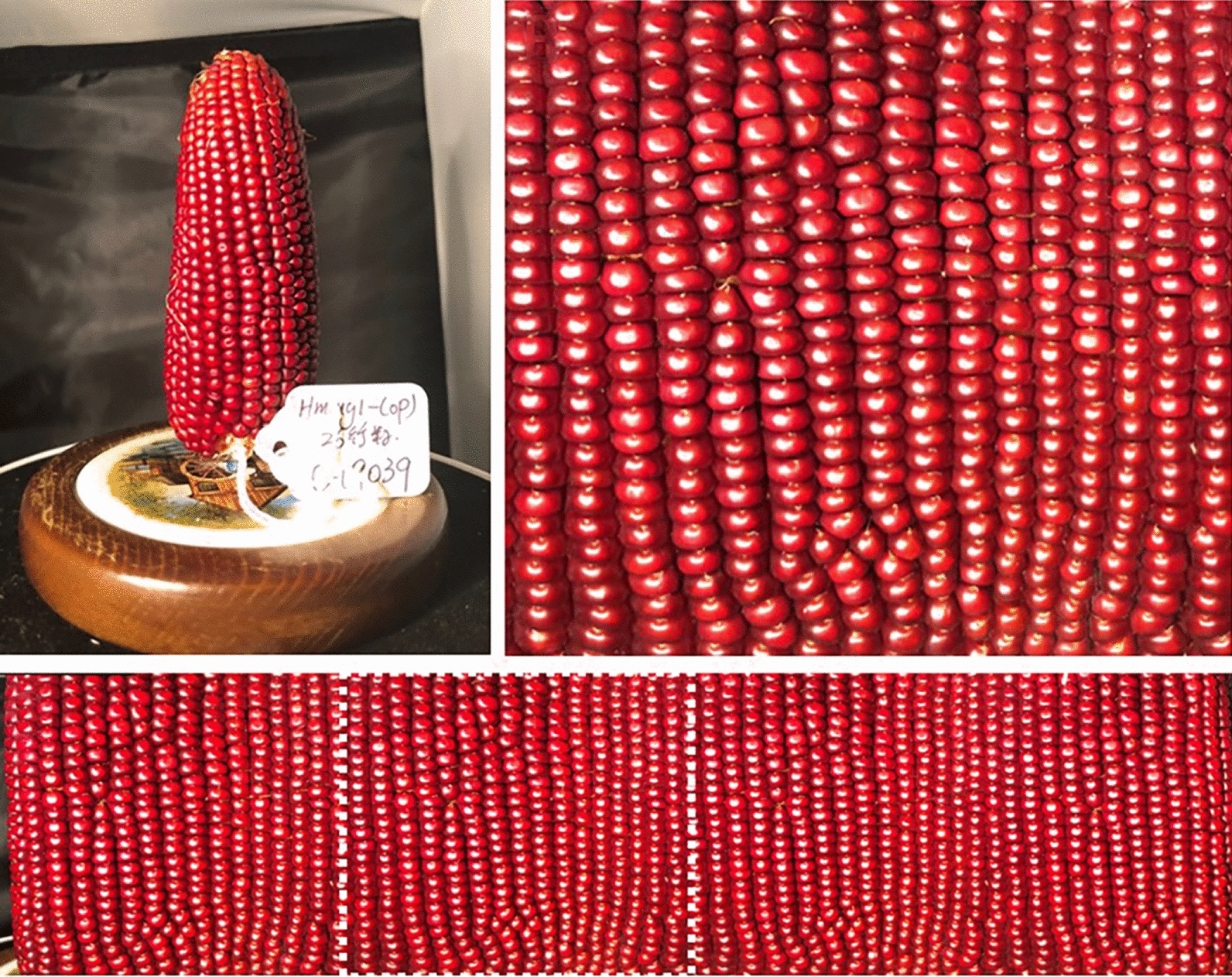
An example of fully prepared panoramic with one full revolution using 16 counted rows. The corn ear (upper L), one full revolution (upper R), and the raw panoramic image with one revolution marked (lower panel)

A raw panorama image is taken when the turn table turns for at least one revolution as a continuous array and the image is cropped to include exact one full revolution of the corn ear (Figs. 8, 9). By using a landmark on the corn ear and cropping the image when the landmark is recognized again, one full revolution is recorded (Fig. 8). Alternatively, the image can be cropped to contain exact one full revolution of the corn ear by counting a specific number of rows. Figure 9 shows an example of counting 16 rows for obtaining one revolution. Once one full revolution of the corn ear is present, the image is fully prepared for kernel quantification.

### Image analysis

#### Total kernel count using binary image analysis

**Fig. 10:**
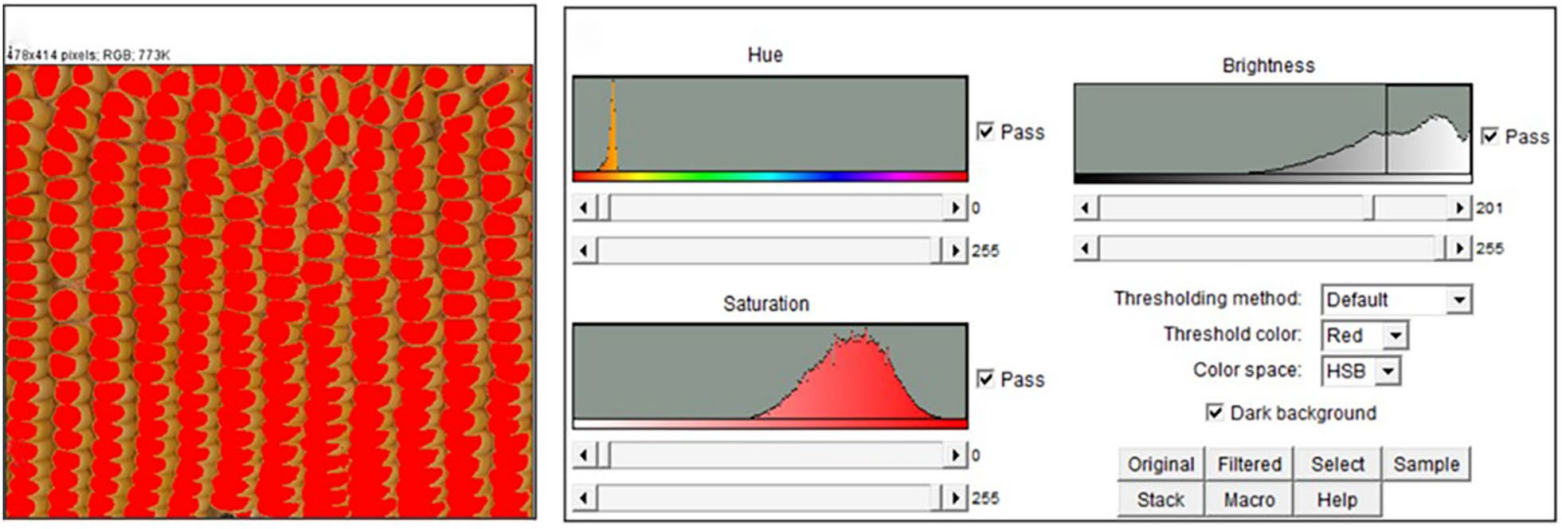
Selecting kernels using Color Threshold in ImageJ. Image after being Color Threshold-ed in ImageJ (L) and an example showing the defaults of color adjustment (R): Hue, Saturation, and Brightness

**Fig. 11:**
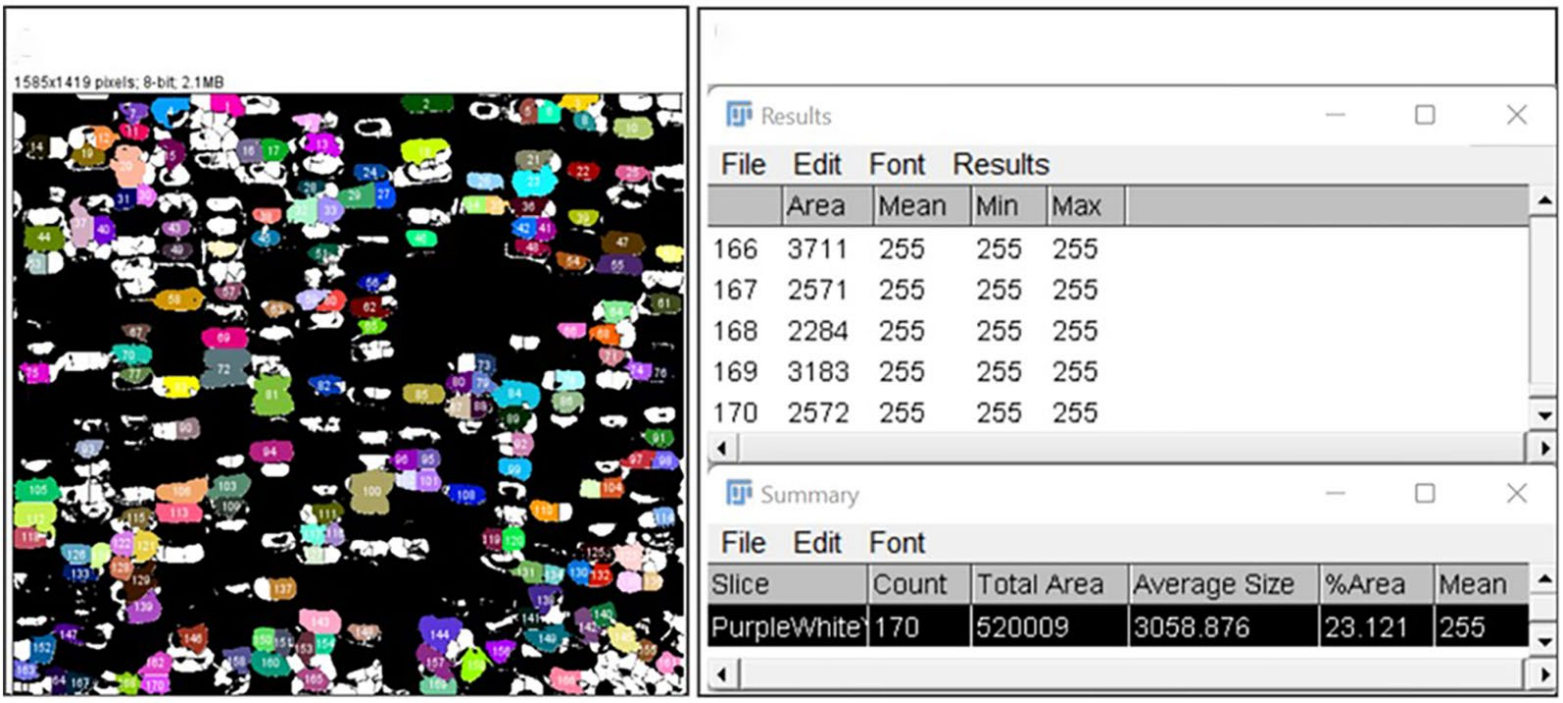
An example of 170 purple kernel count using Analyze Particle of ImageJ. Results of Analyze Particle with particles numbered (L) and Summary of counting results showing 170 kernels (R)

The first step to count the total kernel in the prepared panorama images is changing the image from color to binary using the ImageJ functions Image–Adjust–Color Threshold to select only the kernels (Fig. 10) and Process–Binary–Make Binary to obtain a black and white image (Fig. 2). A balance is to be found when adjusting Color Threshold to keep darker kernels from blending together and lighter kernels from washing out. After changing the image to binary, Process–Binary–Watershed the image to separate any kernels that have blended together or Process–Binary–Fill Holes of the areas that have the kernels washed out. Subsequently use the Analyze Particle function under the analyze menu to count the number of kernels in the image (Fig. 2). It is recommended to eliminate any noise in the image by restricting the size of the particles to be counted to be above 50-to-60-pixel. Figure 11 demonstrates the result of Analyze Particles with analyzed particles highlighted and numbered, giving a purple kernel count of 170. When analyzing a batch of images with similar properties, a macro is written to process a folder of prepared images, from adjusting the image to particle count (Additional file 4). Percent accuracy is calculated as $Accuracy = \frac{{\left| {x_{{\text{m}}} - x_{{\text{a}}} } \right|}}{{x_{{\text{m}}} }}$ where $x_{{\text{m}}}$ is manual count and $x_{{\text{a}}}$ is ImageJ AI count.

#### Segmented kernel count using the Food Color Inspector

**Fig. 12:**
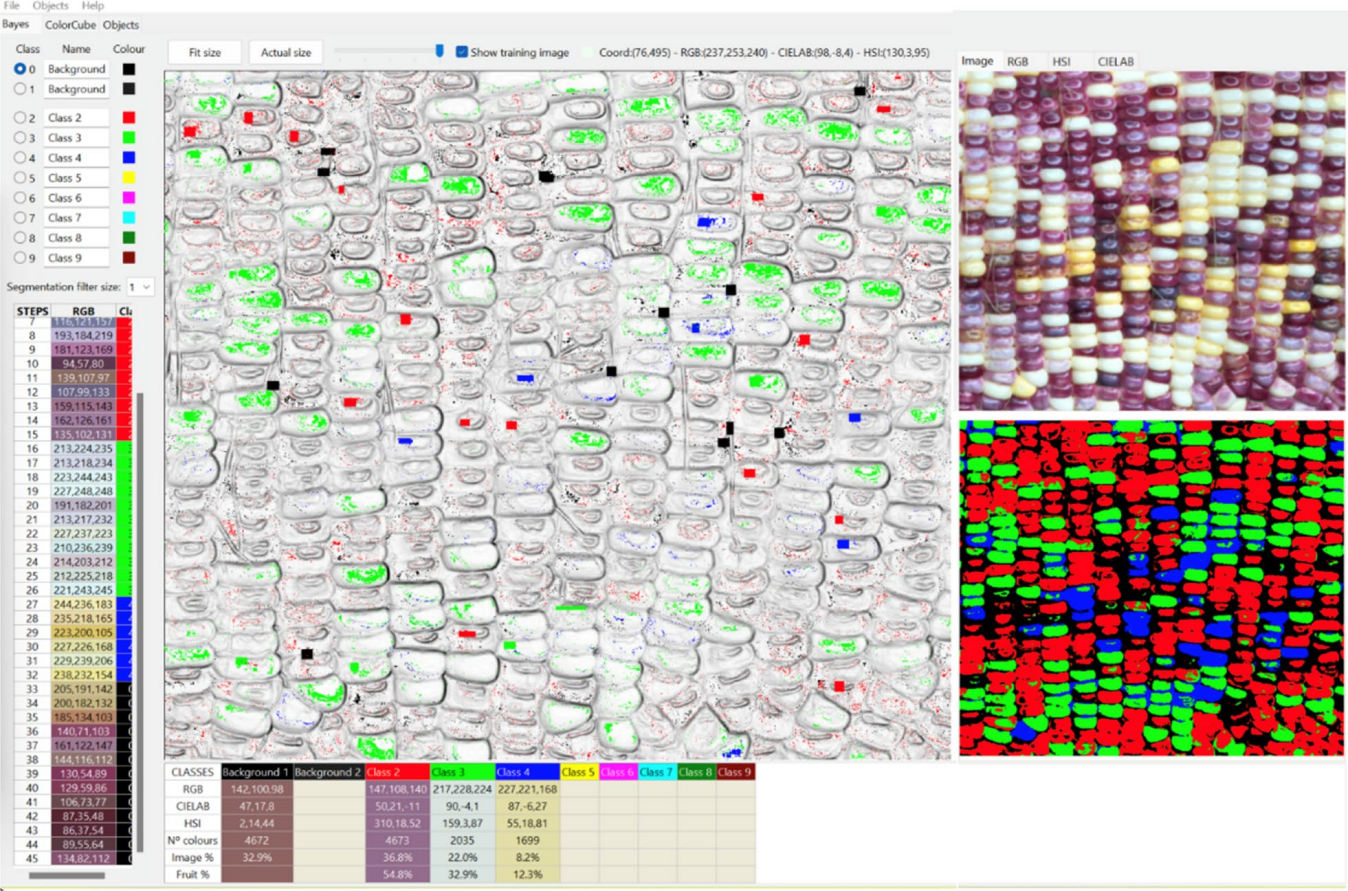
The Food Color Inspector window showing how to train the classifier using examples of the gaps between kernels as the background class, purple kernels as class 2, white kernels as class 3, and yellow kernels as class 4 with the original image and segmented image shown on the right

Food Color Inspector was used to segment the image of hybrid corn ears (http://www.cofilab.com/portfolio/food-color-inspector/). The prepared image of mixed patterned corn ear sample 1 as shown in Fig. 4 was opened in Food Color Inspector and the training was done on a few representative samples of the purple kernels, white kernels, yellow kernels, and the gaps between the kernels as background (Figs. 2, 12). Multiple categories can be made using the Food Color Inspector classifier, but the more categories, the more time the software will take to differentiate them. The obtained segmented image was input into ImageJ, color-thresholded using the default of HSB color space to obtain the image of total kernels with kernels counted as outlined in the previous section, and Color–Split Channels to obtain individual segmented images of yellow kernels, purple kernels, and white kernels (Figs. 2, 5, Additional file 5). The adjusted image was converted to binary, Fill Holes or Watershed as needed and had the particles counted using Analyze Particles (Additional file 5). To obtain a more accurate count, the size of the particles to be counted need to be adjusted for different images (Fig. 2). The size of the particles to be counted was 100-infinity pixel^2^ for sample 3 in Figs. 6 and 7 for total kernel and starch kernel counts, 350-infinity and 300-infinity pixel^2^ for sticky and sweet kernel counts, respectively. The training was subsequently run on an image of mixed patterned corn ear sample 2 as shown in Fig. 4 and sample 4 in Fig. 6 to test its effectiveness (Fig. 2). Once the trained classifier is executed, the segmented image is created (Figs. 2, 4, 6). The Food Color Inspector works very well for batch segmentation of images, where one can train a classifier, save it, and then apply it to images that have similar categories. Similarly, the segmented images of the validation samples were processed by Split Channels, converted to binary, and had particles counted (Fig. 2). The size of particles to be counted was 1800-infinity pixel^2^ for sample 2 in Figs. 4, 5. While the size of particles to be counted was 1000-infinity pixel^2^ for sample 4 kernel counts in Figs. 6, 7 except 1800-infinity pixel^2^ for sticky kernel count.

## Supplementary Information

**Additional file 1.** Examples of dried sticky corns (L), a sweet corn (M), and a starch corn (R).

**Additional file 2.** An example dried hybrid corn ear resulting from a sweet × sticky cross (selfed) whole view (L), panoramic prepared image (M), and side-view (R). Circled in green: starch corn, circled in magenta: sticky corn, and circled in white: sweet corn.

**Additional file 3.** An example panoramic image of tree bark pattern taken by the Corn360 system.

**Additional file 4.** An example of a macro with the steps to process a batch of images to count the total number of kernels in a panorama image of a corn ear.

**Additional file 5.** An example of a macro for batch counting the number of kernels in the Food Color Inspector segmented image: counting total kernels, splitting into separate segmented images, counting purple kernels, counting white kernels, and counting yellow kernels.

## Data Availability

The datasets generated and analyzed during the current study are not publicly available due to the corn varieties currently under selection for breeding purpose but are available from the corresponding author on reasonable request.
