# Supplementary figures and images for: Corn360: a method for quantification of corn kernels
Source: Plant Methods. 2023 Mar 9;19:23. doi: 10.1186/s13007-023-00995-2 (PMC9996904; doi:10.1186/s13007-023-00995-2)

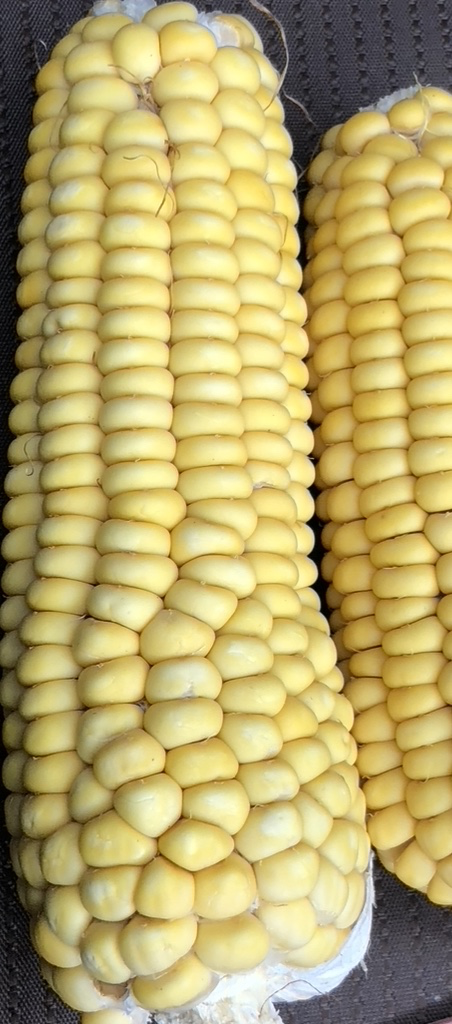

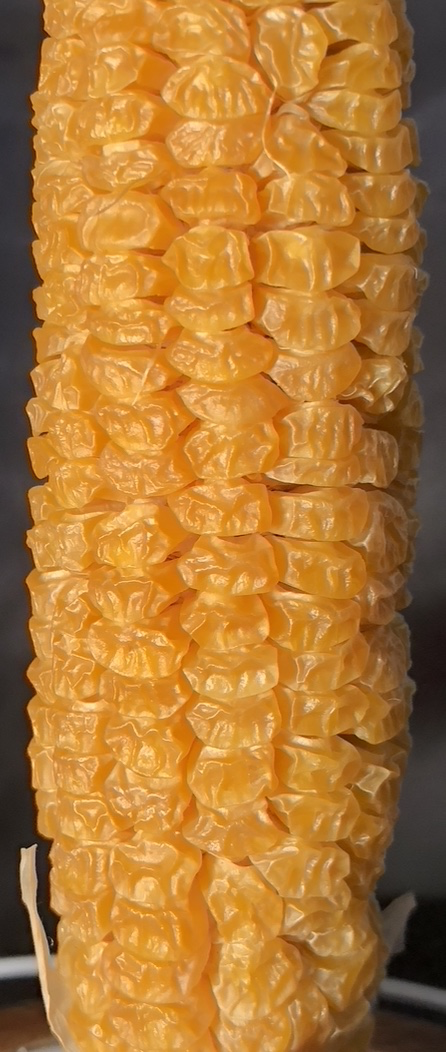

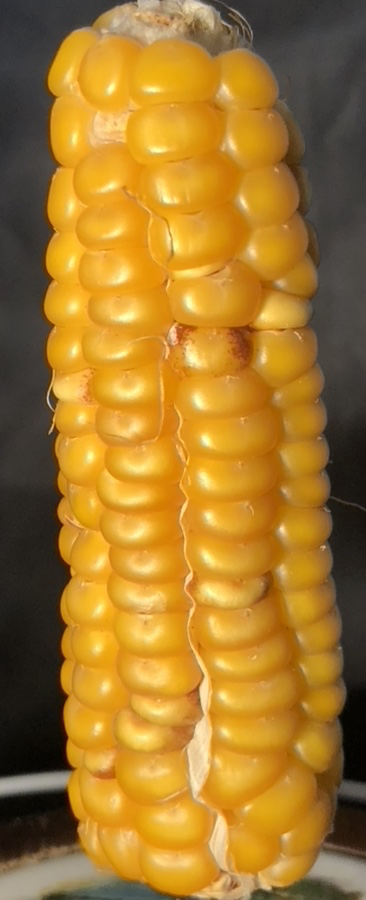


**Additional file 1.** Examples of dried sticky corns (L), a sweet corn (M), and a starch corn (R).

Supplement: Supplementary file 1 — Additional file 1. Examples of dried sticky corns (L), a sweet corn (M), and a starch corn (R). [file 13007_2023_995_MOESM1_ESM.docx]

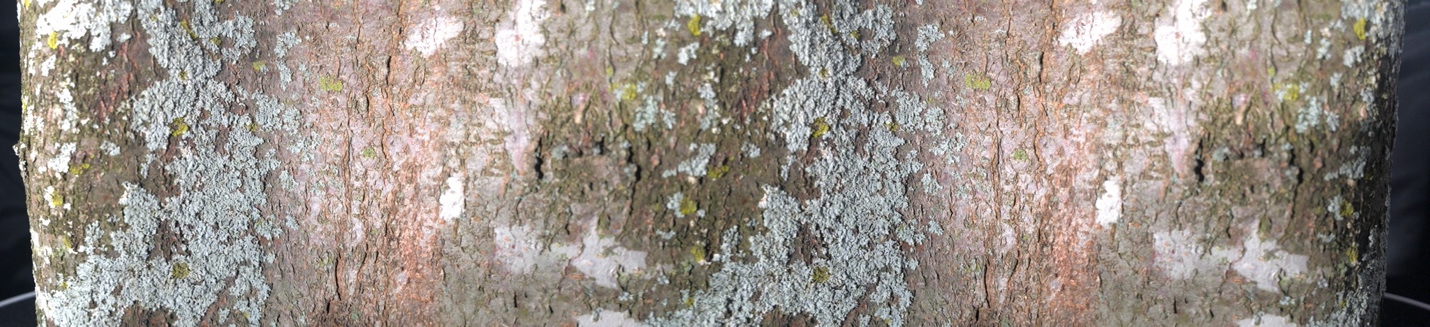


**Additional file 3.** An example panoramic image of tree bark pattern taken by the Corn360 system.

Supplement: Supplementary file 3 — Additional file 3. An example panoramic image of tree bark pattern taken by the Corn360 system. [file 13007_2023_995_MOESM3_ESM.docx]
