# Supplementary material for: Corn360: a method for quantification of corn kernels
Source: Plant Methods. 2023 Mar 9;19:23. doi: 10.1186/s13007-023-00995-2 (PMC9996904; doi:10.1186/s13007-023-00995-2)
Supplement: Supplementary file 2 — Additional file 2. An example dried hybrid corn ear resulting from a sweet × sticky cross (selfed) whole view (L), panoramic prepared image (M), and side-view (R). Circled in green: starch corn, circled in magenta: sticky corn, and circled in white: sweet corn. [file 13007_2023_995_MOESM2_ESM.docx]

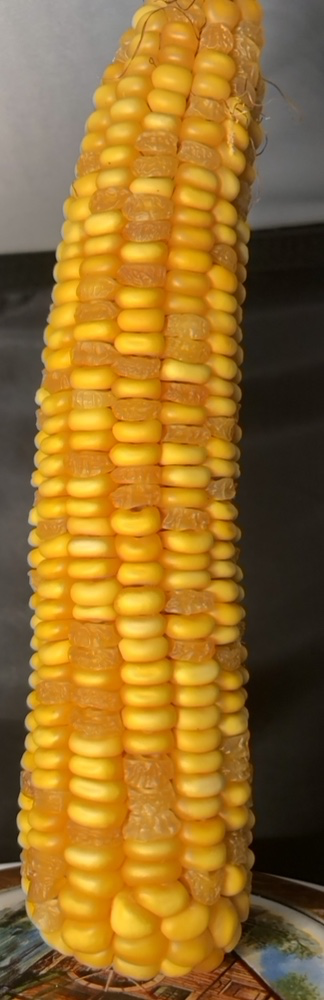

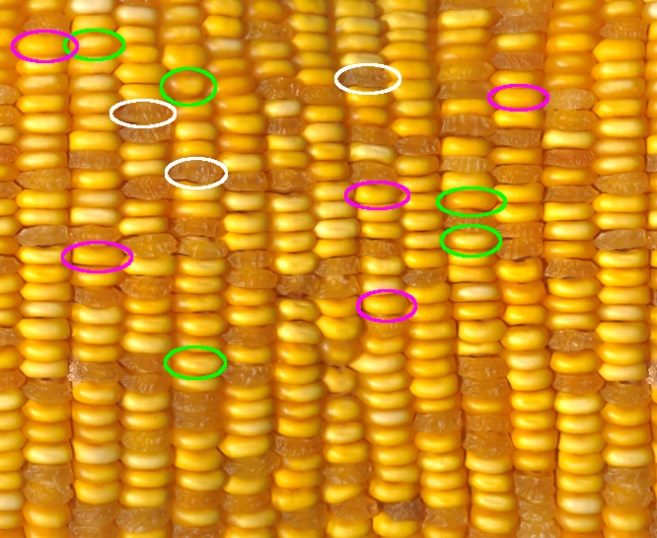

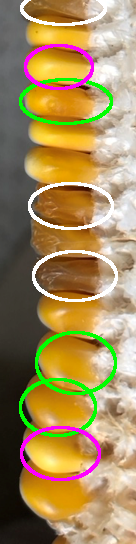


**Additional file 2.** An example dried hybrid corn ear resulting from a sweet × sticky cross (selfed) whole view (L), panoramic prepared image (M), and side-view (R). Circled in green: starch corn, circled in magenta: sticky corn, and circled in white: sweet corn.
