# Supplementary material for: Corn360: a method for quantification of corn kernels
Source: Plant Methods. 2023 Mar 9;19:23. doi: 10.1186/s13007-023-00995-2 (PMC9996904; doi:10.1186/s13007-023-00995-2)
Supplement: Supplementary file 4 — Additional file 4. An example of a macro with the steps to process a batch of images to count the total number of kernels in a panorama image of a corn ear. [file 13007_2023_995_MOESM4_ESM.docx]

**Additional file 4.** An example of a macro with the steps to process a batch of images to count the total number of kernels in a panorama image of a corn ear.

open("C:/Users/preppedimage.jpeg");

run("Color Threshold...");

//setThreshold(255, 255);

setOption("BlackBackground", false);

run("Convert to Mask");

run("Watershed");

run("Fill Holes");

run("Analyze Particles...", "size=60-Infinity display clear summarize");
