# Supplementary material for: Corn360: a method for quantification of corn kernels
Source: Plant Methods. 2023 Mar 9;19:23. doi: 10.1186/s13007-023-00995-2 (PMC9996904; doi:10.1186/s13007-023-00995-2)
Supplement: Supplementary file 5 — Additional file 5. An example of a macro for batch counting the number of kernels in the Food Color Inspector segmented image: counting total kernels, splitting into separate segmented images, counting purple kernels, counting white kernels, and counting yellow kernels. [file 13007_2023_995_MOESM5_ESM.docx]

**Additional file 5**. An example of a macro for batch counting the number of kernels in the Food Color Inspector segmented image: counting total kernels, splitting into separate segmented images, counting purple kernels, counting white kernels, and counting yellow kernels.

open("C:/Users/PurpleWhiteYellowPrepped_segmented.png ");

run("Color Threshold...");

run("Close");

//setThreshold(255, 255);

setOption("BlackBackground", false);

run("Convert to Mask");

run("Fill Holes");

run("Watershed");

run("Analyze Particles...", "size=1800-Infinity show=[Overlay Masks] display clear summarize overlay");

close();

open("C:/Users/PurpleWhiteYellowPrepped_segmented.png ");

run("Split Channels");

selectWindow("PurpleWhiteYellowPrepped_segmented.png (blue)");

saveAs("Tiff", "C:/Users/PurpleWhiteYellowPrepped_segmentedCountedYellow.tif");

close();

saveAs("Tiff", "C:/Users/PurpleWhiteYellowPrepped_segmentedCountedWhite.tif");

close();

saveAs("Tiff", "C:/Users/PurpleWhiteYellowPrepped_segmentedCountedPurple.tif");

close();

open("C:/Users/PurpleWhiteYellowPrepped_segmentedPurple.png ");

run("Options...", "iterations=1 count=1 black do=Nothing");

run("Convert to Mask");

run("Watershed");

run("Analyze Particles...", "size=1800-Infinity show=[Overlay Masks] display clear summarize overlay");

saveAs("Tiff", "C:/Users/PurpleWhiteYellowPrepped_segmentedCountedPurple.tif");

close();

open("C:/Users/PurpleWhiteYellowPrepped_segmentedCountedWhite.tif");

run("Fill Holes");

run("Analyze Particles...", "size=1800-Infinity show=[Overlay Masks] display clear summarize overlay");

saveAs("Tiff", "C:/Users/PurpleWhiteYellowPrepped_segmentedCountedWhite.tif");

close();

open("C:/Users/PurpleWhiteYellowPrepped_segmentedCountedYellow.tif");

run("Fill Holes");

run("Watershed");

run("Analyze Particles...", "size=1800-Infinity show=[Overlay Masks] display clear summarize overlay");

saveAs("Tiff", "C:/Users/yinxx/OneDrive/Desktop/Postdoc/corn360/PurpleWhiteYellowPrepped_segmentedCountedYellow.tif");

close();
